# Hemodynamic changes and neuronal damage detected by 9.4 T MRI in rats with chronic cerebral ischemia and cognitive impairment

**DOI:** 10.1002/brb3.2642

**Published:** 2022-06-10

**Authors:** Minghua Sun, Liangmiao Wu, Guangying Chen, Xukai Mo, Changzheng Shi

**Affiliations:** ^1^ Medical Imaging Center The First Affiliated Hospital of Jinan University No 613 Huangpu Dadao West Guangzhou People's Republic of China; ^2^ Department of Radiology The Fuyang Hospital Affiliated to Anhui Medical University Fuyang People's Republic of China; ^3^ The Engineering Research Center of Medical Imaging Artificial Intelligence for Precision Diagnosis and Treatment Guangzhou Guangdong Province People's Republic of China; ^4^ Institute of New Drug Research, International Cooperative Laboratory of Traditional Chinese Medicine Moderation and innovative Drug Development of Chinese Ministry of Education Jinan University College of Pharmacy Guangzhou People's Republic of China; ^5^ Department of Neurology and Stroke Center The First Affiliated Hospital of Jinan University Guangzhou China

**Keywords:** bilateral common carotid artery occlusion, cerebral hypoperfusion, dementia, magnetic resonance diffusion tensor image, astrocyte

## Abstract

**Introduction:**

The bilateral common carotid artery occlusion (BCCAO) rat model is an ideal animal model for simulating the pathology of chronic brain hypoperfusion in humans. However, dynamic changes in neuronal activity, cellular edema, and neuronal structural integrity in vivo after BCCAO have rarely been reported. The purpose of this study is to use a 9.4 T MRI to explore the pathophysiological mechanisms of vascular dementia.

**Materials and Methods:**

Twelve Sprague–Dawley (SD) rats were randomly divided into two groups: the sham group and the model group (*n* = 6 for each group). Rats were subjected to MRI using T2*WI, diffusion tensor imaging (DTI), and DWI sequences by MRI at the following six time points: presurgery and 6 h, 3 days, 7 days, 21 days, and 28 days postsurgery. Then, the T2*, fractional anisotropy (FA), and average apparent diffusion coefficient (ADC) values were measured in the bilateral cortices and hippocampi. After MRI scanning, all rats in both groups were subjected to the Y‐maze test, novel object recognition test, and open‐field test to assess their learning, memory, cognition, and locomotor activity.

**Results:**

The T2*, FA, and ADC values in the cerebral cortex and hippocampus decreased sharply at 6 h after BCCAO in the model group compared with those of the sham group. By Day 28, the T2* and ADC values gradually increased to close to those in the sham group, but the FA values changed little, and the rats in the model group had worse learning, memory, and cognition and less locomotor activity than the rats in the sham group.

**Conclusions:**

The BCCAO is an ideal rat model for studying the pathophysiological mechanisms of vascular dementia.

## Graphical abstract

BCCAO MRI Dementia

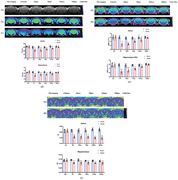

## 1 INTRODUCTION

The rat model of permanent bilateral common carotid artery occlusion (BCCAO) has been widely used in experimental research to simulate the pathological process of vascular dementia in humans (Hazalin et al., 2020). The primary pathophysiological changes associated with BCCAO are decreased cerebral blood flow, free radicals and inflammatory reactions, neuronal apoptosis in the cortex and hippocampus, glial cell proliferation (Vicente et al., 2009), and cognitive memory impairment (Xi et al., 2014). However, dynamic changes in neuronal activity, cellular edema, and neuronal structural integrity in vivo after BCCAO have rarely been reported.

Paramagnetic deoxyhemoglobin in the blood can shorten the T2* relaxation time (Wedegärtner et al., 2010). Based on this principle, T2* is often used to evaluate the metabolic status of brain tissue (Chavhan et al., 2009). Within a few minutes of cerebral hypoperfusion, ischemic cascade reactions such as cell energy failure (Busza et al., 1992), cell membrane Na^+^‐K^+^‐adenosine triphosphatase pump inhibition (Qiao et al., 2002), and cell membrane depolarization (de Crespigny et al., 1999) appear. All these reactions are related to the decrease in the apparent diffusion coefficient (ADC) in brain tissue. If the hypoperfusion status of brain tissue is restored to normal in a timely manner, the ADC value of the brain tissue increases (Sotak, 2004). Fractional anisotropy (FA), as a common quantitative index of diffusion tensor imaging (DTI), reflects the diffusion characteristics of water molecules in tissues, which is helpful in investigating microstructural abnormalities (Yoon et al., 2006).

In this study, we observed the dynamic changes in the T2*, ADC, and FA values after cerebral ischemia. We combined the results from the Y‐maze experiment, the novel object recognition (NOR)test and open‐field tests to study the effects of the dynamic changes in cerebral hemodynamics, neuronal edema, and neuronal damage after BCCAO in rats.

## 2 MATERIALS AND METHODS

### 2.1 Animals and experimental groups

Male Sprague–Dawley (SD) rats (weight: 200–220 g, mean: 210.7 ± 4.0 g at the beginning of the study; 65 days old) were provided by the Medical Experiment Animal Center of Guangdong Province. Twelve rats were randomly divided into two groups (*n* = 6, each group): the sham group and the model group. All rats were housed in cages under controlled temperature (23.2 ± 1.3℃) and humidity (60.4 ± 5.1%) conditions with a constant 12‐h light/12‐h dark cycle. All rats were fed according to the relevant feeding management regulations of the Experimental Animal Management Center of Jinan University. The protocol was approved by the competent ethics committees at Jinan University.

### 2.2 Preparation of the BCCAO model

The rats in the model group were fasted for 8–10 h one night before the operation and were allowed to drink freely. The rats in the model group were anesthetized with pentobarbital sodium (30 mg/kg, intraperitoneal). Once they failed to respond to a painful stimulus, the hair in the middle of the rats’ necks was shaved and disinfected. The median skin was cut, and the bilateral common carotid arteries and the adjacent vagus nerves were carefully separated. Then, each side of the common carotid artery was ligated with two 3‐0 sutures.

### 2.3 Magnetic resonance imaging

**FIGURE 1:**
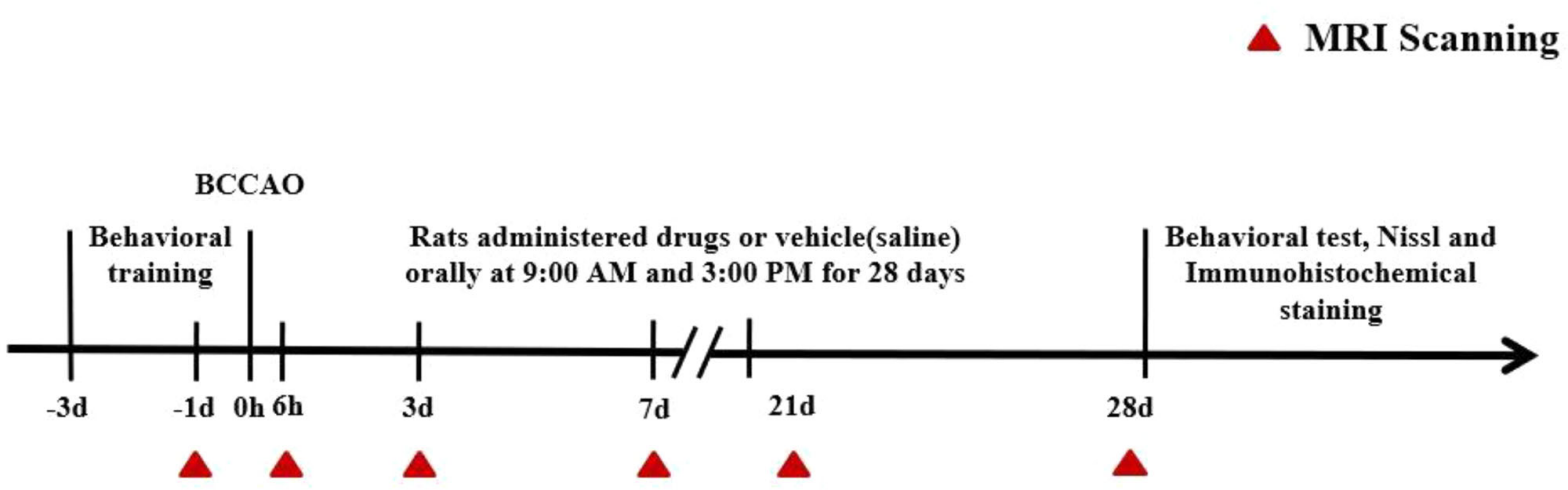
Animal experiment workflows

Rats were imaged using a 9.4 T Bruker Biospec 94/30 USR at Jinan University. Signals were generated and received by an 86‐mm‐ID orthogonal volume transmitter combined with a 20‐mm‐diameter single‐ring surface coil (Bruker, Germany) placed on the top of the rat skull. Rats were subjected to MRI (including T2star_mapping_MGE (T2*WI), diffusion‐weighted imaging (DWI), and DTI sequences at the following six time points (Figure 1): presurgery and 6 h, 3 days, 7 days, 21 days, and 28 days postsurgery. The scanning for each sequence are shown in Table 1.

**TABLE 1:** Magnetic resonance sequence scanning protocols

	T2*WI	DWI	DTI
Repetition time/echo time (ms)	1050.000 /3.500	3000.000/20.000	3000.000/20.000
Averages	2	2	8
Repetition	1	1	1
Flip angle	50.0°	90.0°	90.0°
Echo images	9	–	–
Slices	19	19	19
Slice thickness (mm)	1.000	1.000	1.000
Image size	256 × 256	96 × 96	108 × 96
Field of view (mm)	25 × 25	25 × 25	25 × 25
Dummy duration (ms)	2100.000	3000.000	3000.000
Resolution	0.098 × 0.098	0.260 × 0.260	0.231 × 0.260
Slice gap (mm)	0.300	0.300	0.000
Slice distance (mm)	1.300	1.300	1.000
Bandwidth	69,444.4	340,909.1	33,3333.3
*b* value (s/mm^2^)	–	800	650

**FIGURE 2:**
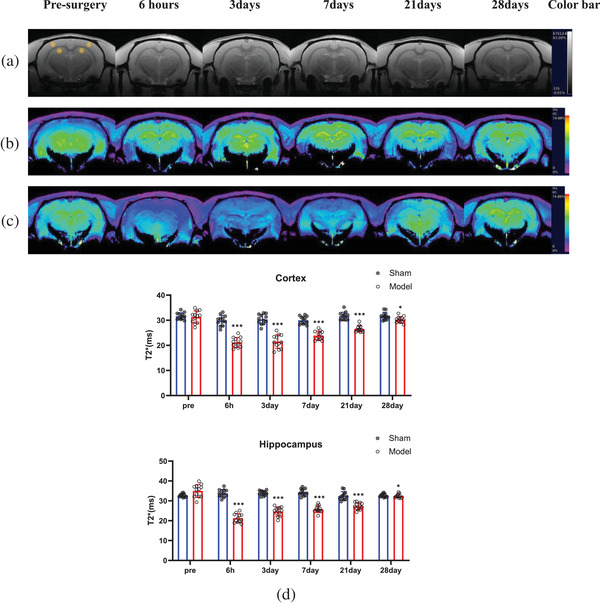
Magnetic resonance imaging (MRI) images showing changes in T2^*^ in the cortex and hippocampus of rats. The MRI scanning time points from left to right are presurgery and 6 h, 3 days, 7 days, 21 days, and 28 days after BCCAO. (a) Gray images are from rats in the model group. The yellow circle in the leftmost image represents the area where the T2^*^ value was measured. (b) Color images are from rats in the sham group. Areas in yellow in T2^*^WI images represent the strongest T2* signal, areas in violet reflect the weakest T2* signal, and the green or blue color is an intermediate signal. (c) Color images are from rats in the model group. The colors have the same meaning as in (b). (d) Histogram showing quantitative results of T2^*^ at different time points after BCCAO. In the cortex and hippocampus, the T2^*^ values in the sham group were not significantly different at different MRI scanning time points (*p* < 0.001). In the model group, T2^*^ values immediately decreased 6 h after BCCAO (*p* < 0.001) and then gradually recovered from the third day. On the 28th day, the T2^*^ value was close to the preoperative level. ^*^ *p* < 0.05, ^**^ *p* < 0.01, ^***^ *p* < 0.001

T2*, FA, and ADC images were automatically generated by the Paravision 360 V1.1 workstation. The T2*, ADC, and FA values were measured in the following four regions: the bilateral cortices and the bilateral hippocampi (Figure 2a). The area of the regions of interest (ROIs) was approximately 1.1 mm^2^. Each ROI was measured three times, and the average value of each ROI was taken as the final value.

### 2.4 Y‐maze test

The Y maze test was used to determine the spatial working memory of the rodents. The test was performed according to a previously described method (Jin et al., 2017) the day after MRI scanning. The time spent in the three arms of the maze and the number of shuttles were both recorded by a camera above the Y‐maze. Learning and memory functions were evaluated by the residence time discrimination index. The specific calculation formula used was novel arm time/total explored time.

### 2.5 Novel object recognition test

The NOR test is often used to study the cognitive ability of animals. It was performed according to a previously described method (Karasawa et al., 2008) the day after the Y‐maze test. During the training and testing, tracking of the rats’ head movements was recorded by a camera above the open box, and the cognitive and memory abilities of the rats were evaluated based on the exploration time of novel objects (NT) and old objects (or familiar objects, FT). The discrimination index was used to quantify the preference of the experimental animals for novel and familiar objects. The discrimination index was calculated by the formula: (NT − FT)/(NT + FT).

### 2.6 Open‐field test

The open‐field test is a method for evaluating the locomotor and exploratory behavior of rodents in new environments. The movement of the rats in the field was tracked by a camera directly above the open chamber and recorded for 5 min. Smart v3.0 software (Panlab) was used to analyze the total moving distance and movement speed of the rats.

### 2.7 Tissue Preparation

After the behavioral tests, the rats were sacrificed by deep anesthesia with pentobarbital sodium (60 mg/kg, intraperitoneal). The thoracic cavity was opened, a 50‐mL syringe needle was inserted from the apex of the heart to the aortic arch, the right atrial appendage was cut open, and precooled PBS (approximately 200 mL) was perfused until the viscera turned white and the blood from the atrial appendage was clear or did not exhibit blood staining. Then, the brains were removed, quickly frozen in liquid nitrogen, and placed in an −80°C freezer for long‐term storage.

### 2.8 Nissl Staining

The removed brain tissue was fixed in 4% paraformaldehyde. Staining was performed according to a previously described method (Jin et al., 2017). Cell counting was performed and analyzed by an experimenter blinded to the groups using ImageJ software (Bethesda, MD) at 20× magnification.

### 2.9 Immunohistochemical staining

Staining was performed according to a previously described method (Chen et al., 2020). The cell counting method was the same as that used for Nissl staining.

### 2.10 Statistical analysis

In the experiment, MRI results, behavioral test results, and immunostaining cell counts were analyzed in a double‐blind manner. All data were plotted with GraphPad Prism 8.0.2 and analyzed using SPSS 23.0 statistical software. Normality assumptions for all data were verified by the Shapiro–Wilk test, with *p* > 0.05 indicating that the data obeyed a normal distribution. Independent‐samples *t*‐tests were used to compare the measured data between rats in the sham group and the model group according to the specific situation. The data are expressed as the mean ± standard deviation, with *p* < 0.05 representing a significant difference.

## 3 RESULTS

### 3.1 Alterations in T2* values in the cortices and hippocampi of rats in the sham group and the model group at different time points

In the T2*WI grayscale images (Figure 2a), no infarction was found in any brain tissue at any time point before or after BCCAO in either group. During T2*WI pseudoimaging, the colors of the brain tissue of rats in the sham group at all time points and in the model group before surgery were mostly yellow (indicating a strong signal) (Figure 2b). Six hours after surgery, the signal in most regions turned blue (indicating a weak signal) or violet (the weakest signal) in the model group, and the blue or violet signals persisted until the seventh day. After that, some areas gradually returned to green (intermediate signal between yellow and blue) or yellow (Figure 2c).

The quantitative results values followed a normal distribution based on the Shapiro–Wilk test (Figure 2d). There were no significant differences in the T2* values in the bilateral cortices and hippocampi of rats in the sham group at the above six time points and in the model group at the preoperative time point (*p* < 0.001). In the model group, the T2* value in the bilateral cortices (21.13 ± 1.98) and hippocampi (21.31 ± 2.22) 6 h after surgery was lower than that (cortex: 29.87 ± 2.07; hippocampus: 33.79 ± 1.95) before surgery (*p* < 0.001). Then, the T2* value in the bilateral cortices and hippocampi of the rats gradually increased. By the 28^th^ day, the T2* value (cortex: 30.24 ± 1.25; hippocampus: 31.83 ± 1.10) was very close to that of the sham group (cortex: 31.65 ± 1.50; hippocampus: 33.20 ± 1.77) (cortex: *p* = 0.020, hippocampus: *p* = 0.033).

### 3.2 Differences in ADC values in the cortices and hippocampi of rats in the sham group and the model group at different time points

**FIGURE 3:**
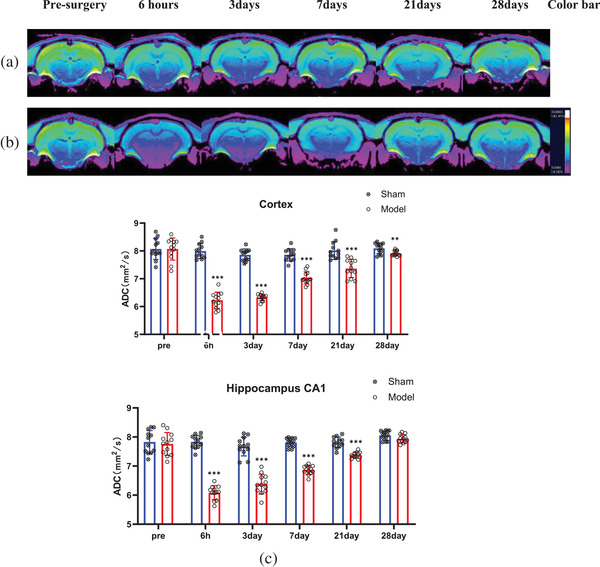
MRI images showing changes in ADC values in the cortex and hippocampus of rats. The MRI scanning time points from left to right are presurgery and 6 h, 3 days, 7 days, 21 days, and 28 days after BCCAO. The regions and areas measured were the same as those with the T2*WI sequence. (a) Color images from rats in the sham group. (b) Color images from rats in the model group. The colors have the same meaning as in (b) for the T2^*^WI images. (c). Histogram showing quantitative ADC results at different time points after BCCAO. In the cortex and hippocampus, the ADC values in the sham group showed no significant differences at different MRI scanning time points (*p* < 0.001). In the model group, ADC values immediately decreased 6 h after BCCAO (*p* < 0.001) and then gradually recovered from the third day. On the 28th day, the ADC value was close to the preoperative level. ^*^ *p* < 0.05, ^**^ *p* < 0.01, ^***^ *p* < 0.001

In the ADC images, most areas of the bilateral cortices and hippocampi in the sham group at all six time points and in the model group before surgery were yellow (Figure 3a). At 6 h postsurgery, most areas of the cortices and hippocampi were blue or green in the model group (Figure 3b). From the third to the seventh day, the green area became increasingly larger, and the yellow areas appeared on the 21st day. Most of the areas were yellow by the 28th day.

The quantitative ADC values in all regions followed a normal distribution based on the Shapiro–Wilk test (Figure 3c), and there were no significant differences in the ADC values in the bilateral cortices and hippocampi of rats in the sham group at the six time points and in the model group at the preoperative time point. In the model group, the ADC value in the bilateral cortices ((6.23 ± 0.29) mm^2^/s) and hippocampi ((6.08 ± 0.24) mm^2^/s) 6 h after surgery was lower than that (cortex: (8.00 ± 0.27) mm^2^/s, hippocampus: (7.83 ± 0.22) mm^2^/s) before surgery (*p* < 0.001). Then, the ADC values in the above areas gradually increased. On the 28th day, the ADC value (cortex: 7.92 ± 0.10; hippocampus: 7.94 ± 0.14) was close to that of the sham group (cortex: 8.09 ± 0.19; hippocampus: 8.06 ± 0.16) (cortex: *p* = 0.009, hippocampus: *p* = 0.065).

### 3.3 Differences in FA values in the cortices and hippocampi of rats in the sham group and model group at different time points

**FIGURE 4:**
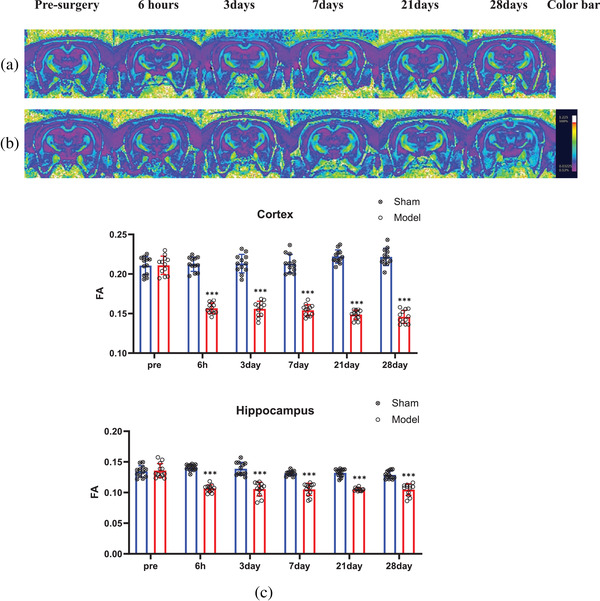
MRI images showing changes in FA values in the cortex and hippocampus of rats. The MRI scanning time points from left to right are presurgery and 6 h, 3 days, 7 days, 21 days, and 28 days after BCCAO. The regions and areas measured were the same as those with the T2*WI sequence. (a) Color images from rats in the sham group. (b) Color images from rats in the model group. Areas in yellow represent the strongest FA signal, and areas in violet reflect the weakest FA signal. With the passage of time, the violet area of the cortex and hippocampus in the model group gradually decreased, but there was no significant change in the violet area in the sham group. (c). Histogram showing quantitative FA results at different time points after BCCAO. The FA value in the cortex and hippocampus in the model group immediately decreased 6 h after BCCAO (*p* < 0.001) and then continued to decrease slightly until the 28th day (*p* < 0.001). ^*^ *p* < 0.05, ^**^ *p* < 0.01, ^***^ *p* < 0.001

In the FA pseudocolor images, all areas of the corpus callosum appeared yellow (the strongest signal), while areas of the cortices and hippocampi were violet (the weakest signal) at all six time points in both groups. The areas of the cortices and hippocampi were similar in the sham group at all six time points and the model group at the preoperative time point (Figure 4a). However, these values were larger than those in the model group at the other five time points (Figure 4b).

The quantitative FA values in all regions followed a normal distribution based on the Shapiro–Wilk test (Figure 4c), and there were no significant differences in the FA values in the bilateral cortices and hippocampi of rats in the sham group at the abovementioned six time points and in the model group at the preoperative time point. Six hours after surgery, the FA values in the cortices (0.1567 ± 0.0067) and hippocampi (0.1067 ± 0.0058) in the model group began to decrease and were lower than those in the sham group (cortex 0.2122 ± 0.0090; hippocampus 0.1408 ± 0.0050) (*p* < 0.001). From the third to the 28th day, the FA values in the bilateral cortices and hippocampi continued to decrease slightly. However, on the 28th day, the FA value (cortex: 0.1458 ± 0.0082; hippocampus: 0.1048 ± 0.0094) in the model group was still lower than that in the sham group (cortex: 0.2216 ± 0.0110; hippocampus: 0.1287 ± 0.0064) (*p* < 0.001).

### 3.4 Comparison of learning and memory abilities of rats before and after surgery

**FIGURE 5:**
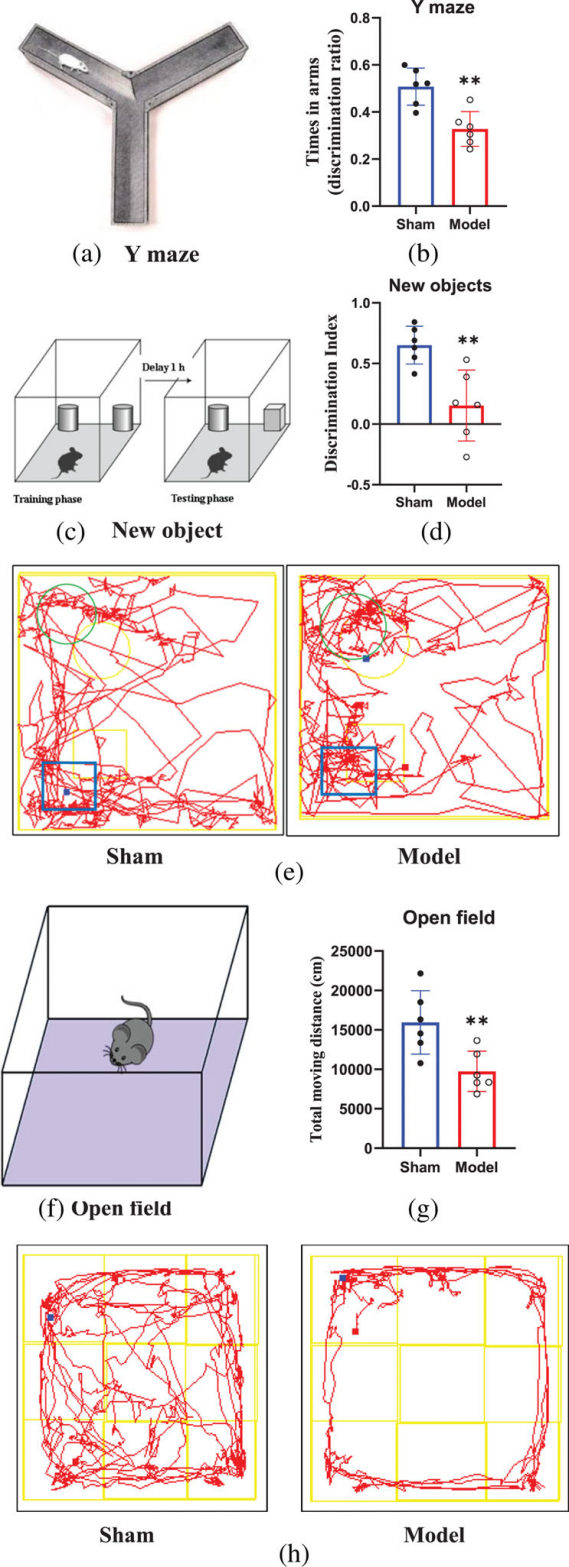
Comparison of learning, memory, cognitive function, and locomotor activity in the rats. Schematic diagram of the new arm in the Y‐maze (a). The residence time discrimination index in the model group was shorter than that in the sham group (b) (*p* < 0.05). Schematic diagram of the NOR experimental equipment (c). The novel object discrimination index of rats in the model group was lower than that of rats in the sham group (d) (*p* < 0.05). The shuttle times of rats in the sham group around the novel object were higher than those around the old object, but those of rats in the model group around the two objects were roughly the same (e). Schematic diagram of the open‐field experiment (f). The total moving distance traveled by rats in the sham group was clearly greater than that traveled by rats in the model group (g) (*p* < 0.05). The red line represents the trajectory of the rat's center of gravity (h). The movement distance and range of rats in the sham group were greater than those in the model group. ^*^ *p* < 0.05, ^**^ *p* < 0.01, ^***^ *p* < 0.001

The Y‐maze test can detect the learning and memory abilities in rodents after hippocampal injury (Sarnyai et al., 2000). As shown in Figure 5b, the discrimination index of the entire residence time in the novel arm was significantly lower in the rats in the model group than in the rats in the sham group (*p* = 0.002).

### 3.5 Comparison of cognitive abilities of rats before and after surgery

NOR testing has been widely used to assess cognitive abilities in rodent experiments (Vidyanti et al., 2020). The quantitative results (Figure 5d) demonstrated that the novel object discrimination index for rats in the model group was lower than that for rats in the sham group (*p* = 0.004).

### 3.6 Locomotor activity of rats before and after surgery

The open‐field test is a classic behavioral experiment that has been widely used to assess rodent locomotor activity (Zuloaga et al., 2015). Figure 5g shows that the total distance traveled by rats in the sham group was clearly greater than that traveled by rats in the model group (*p* = 0.010). Moreover, there was almost no movement of rats in the model group in the central area of the open field.

### 3.7 Changes in neurons in the cortex and hippocampus before and after BCCAO

**FIGURE 6:**
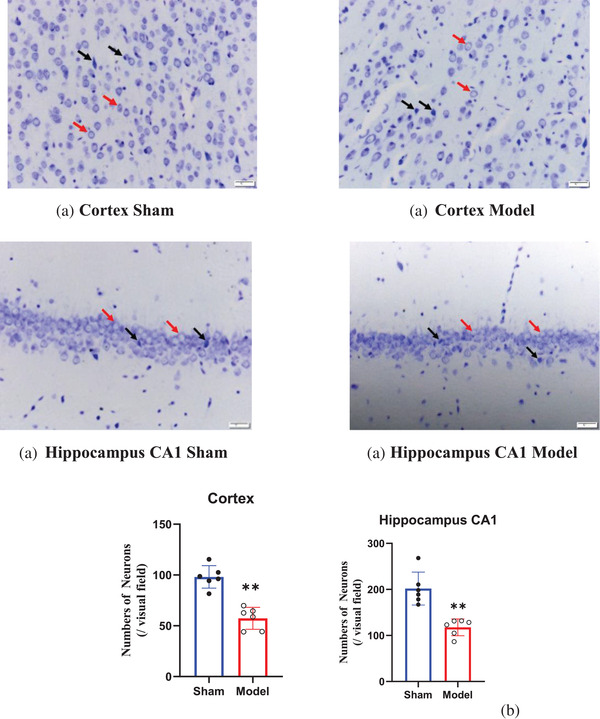
Nissl staining of the cortex and hippocampal CA1 region (0.35 mm^2^) of rats. Normal neurons (red arrow) were arranged in an orderly manner, with small nuclei and weak cytoplasmic staining, and the injured neurons (black arrow) became disorderly, the size of the nucleus increased, and the cytoplasm decreased sharply and was stained dark blue (a). Quantitative results are expressed as the mean ± SD (b) (magnification: 20×). In the cortex and hippocampus, the number of normal cells in the sham group was greater than that in the model group 28 days after BCCAO (*p* < 0.01). ^*^ *p* < 0.05, ^**^ *p* < 0.01, ^***^ *p* < 0.001

Cerebral hypoperfusion can easily lead to neuronal injury, which can impair learning and cognition (Boehm‐Sturm et al., 2017). Figure 6a shows that the cells of normal neurons were arranged in an orderly manner, with small nuclei and weak cytoplasmic staining (red arrow). The normal structure disappeared in damaged neurons; the neurons became disorderly, the size of the nucleus increased, and the cytoplasm decreased sharply and was stained dark blue (black arrow). Compared with the rats in the sham group, the rats in the model group had significantly fewer normal neurons and significantly more injured and degenerated neurons (*p* < 0.001) (Figure 6b).

### 3.8 Alterations in astrocytes in the cortex and hippocampus before and after BCCAO

**FIGURE 7:**
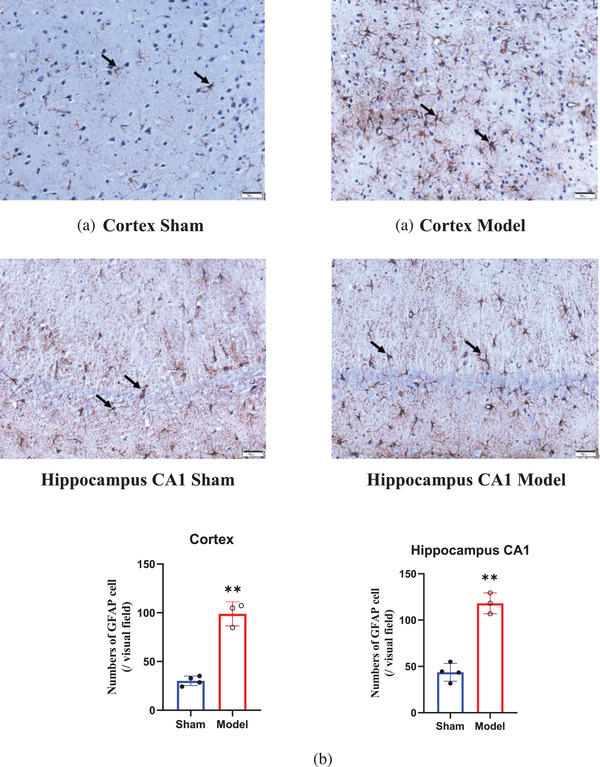
Activated astrocytes in the cortex and hippocampal CA1 region (0.35 mm^2^) of rats. Astrocytes in the cortex and hippocampal CA1 regions are marked with a black arrow (a). Quantitative results are expressed as the mean ± SD (b). (magnification: 20×). The number of astrocytes in the cortex and hippocampal CA1 area of the rats in the model group was greater than that in the model group 28 days after BCCAO (*p*<0.01). ^*^ *p* < 0.05, ^**^ *p* < 0.01, ^***^ *p* < 0.001

Astrocytes are the most widely distributed cells in the mammalian brain and are the largest glial cells (Matsuyama et al., 2020). The number of astrocytes (Figure 7a, black arrow) in the cortex and hippocampal CA1 area of the rats in the model group, compared with the sham group, was increased by nearly three times 28 days after BCCAO (*p* < 0.001) (Figure 7b).

## 4 DISCUSSION

Hypoperfusion of brain tissue is a promoter of many neurological and mental diseases (Ferro et al., 2020). A continuous and moderate decrease in cerebral blood flow (CBF) leads to impairments in memory and cognitive function (Moghaddasi et al., 2017). After BCCAO, CBF in the bilateral anterior cerebral artery and middle cerebral artery decreased immediately (Jing et al., 2015). Amazingly, these ischemic cerebral areas could remain in a hypoperfusion state for a period of time because of the distribution of blood by the complete circle of Willis. In this study, our results revealed that the T2* and ADC values in the cortex and hippocampus of rats experienced a very similar trend after BCCAO, and both values recovered to near‐normal levels on the 28th day. These findings indicate that the brain tissue of rats has a strong compensatory ability related to blood perfusion after BCCAO. However, the FA value did not return to normal, which indicated that the damaged neurons did not completely recover on the 28th day after BCCAO. For this reason, the learning, memory, cognition, and locomotor activity of rats did not recover on the 28th day after BCCAO.

The functional MRI T2*WI sequence has been widely used in experimental and clinical situations to detect tissue activity in vivo (Darcourt et al., 2019). In this study, the T2* values in the cortex (21.13 ± 1.98) and hippocampus (21.31 ± 2.22) in the model group were clearly lower than those (cortex: 29.87 ± 2.07; hippocampus: 33.79 ± 1.95) in the sham group 6 h after BCCAO (*p* < 0.001). This result was due to an increase in the oxygen extraction fraction in ischemic brain tissue, which leads to an increase in deoxyhemoglobin concentrations in cerebral veins and capillaries (Lee et al., 2003). Starting on the third day, the T2* value began to increase (cortex: 21.49 ± 2.71; hippocampus: 24.62 ± 2.27). This indicated that the perfusion of the brain tissue had begun to ameliorate. Several compensatory mechanisms have been reported, including vertebral artery and basilar artery dilation (Jing et al., 2015). The concentration of NO, which has a vasodilating effect, was increased in the blood during cerebral ischemia (Qi et al., 2020) and neovascularization (Jing et al., 2015). On the 28^th^ day, T2* values in the cortex and hippocampus in the model group were very close to those in the sham group.

In the present study, the ADC values in the cortex and hippocampus in the model group significantly decreased 6 h after BCCAO. This suggested that cytotoxic edema in the rat brain cells was due to an insufficient blood supply. As the T2* results indicated, the ADC values in the cortex and hippocampus have increased from the third day to the 28th day. These increases in the ADC values in the brain tissue were not the result of brain tissue evolving into angiogenic edema because no necrotic brain tissue was observed on the T2*WI images at any time point (Figure 2a); this increase was due to the recovery of cerebral perfusion.

In this study, 6 h after BCCAO, the FA values in the bilateral cortices and hippocampi were clearly decreased. This may have been caused by cytotoxic edema of the brain tissue (Wang et al., 2017). FA values in the abovementioned regions continued to decrease slightly at the 3 (cortex: 0.1560 ± 0.0096, hippocampus: 0.1057 ± 0.0112), 7 (cortex: 0.1542 ± 0.0072, hippocampus: 0.1051 ± 0.0099), 21 (cortex: 0.1487 ± 0.0059, hippocampus: 0.1049 ± 0.0025), and 28 days (cortex: 0.1458 ± 0.0082, hippocampus: 0.1048 ± 0.0094) after BCCAO. The reason for this may be that the hypoxia damaged the integrity of the dendrites and axons of the neurons (Mamata et al., 2004), which had not been repaired. In addition, astrocyte activation can cause additional neuronal damage (Zhang et al., 2019) and brain microstructure remodeling, which can lead to an increase in the FA values (Williamson et al., 2021). Therefore, cerebral hypoperfusion both decreases and increases the FA value.

Chronic brain hypoperfusion can lead to cell degeneration and astrocyte activation (Xi et al., 2014). Our study yielded similar results. The number of normal cells dramatically decreased in the cortical and hippocampal CA1 regions of rats in the model group, and the disorderly arrangement and normal structure of injured neurons disappeared by the 28th day after BCCAO. At the same time, astrocytes significantly proliferated. Our quantitative immunohistochemistry results indicated that the number of astrocytes in the cortex and hippocampus in the model group was twice as high as that in the sham group. These results indicated long‐term neuronal damage after BCCAO. It has been reported that damaged hippocampal neurons continue to deteriorate from 4 to 13 weeks after BCCAO (Liu et al., 2006). The histological changes in rat brain tissue may be the pathological basis for the continuous decrease in FA values in brain tissue 28 days after BCCAO, which can also lead to the incomplete recovery of learning, cognition, memory, and behavior. In this study, consistent with neuronal regression, our results showed that the learning, memory, cognition, and locomotor activity of rats were disrupted after BCCAO. The discrimination index of the entire residence time in the Y‐maze, the novel object discrimination index in the NOR test, and the total distance traveled in the open‐field test of rats in the model group were significantly lower for the rats in the model group than for the rats in the sham group. This functional damage has been reported to be the result of a long‐term process (De Jong et al., 1999).

A limitation of this study is the small number of rats in each group. In addition, neurons and glial cells on histological sections and behavior tests were not evaluated at all scanning time points. Moreover, the duration of the experiment was too short, and the pathological changes after BCCAO were assessed after a longer time (e.g., 6, 8, and 12 weeks) may have great clinical significance.

## 5 CONCLUSION

In conclusion, the BCCAO rat model is an ideal animal model for studying the pathophysiological mechanisms of vascular dementia. Multimodal MRI is an effective and convenient tool for noninvasive dynamic observations of cerebral hemodynamics, neuronal edema, and neuronal injury at various stages of chronic cerebral ischemia.

## CONFLICT OF INTEREST

All authors declare no conflict of interest.

## AUTHOR CONTRIBUTIONS

Minghua Sun: conceptualization, writing‑original draft preparation, data curation, validation, writing‑ reviewing, and editing. Liangmiao Wu: methodology, investigation, data curation, writing‑review, and editing. Guangying Chen: methodology, validation, formal analysis. Xukai Mo: conceptualization, software, resources, writing‑review and editing, supervision. Changzheng Shi: investigation, methodology, project administration, resources, supervision, validation. All authors read and approved the final manuscript.

### PEER REVIEW

The peer review history for this article is available at https://publons.com/publon/10.1002/brb3.2642

## References

[brb32642-bib-0001] Boehm‐Sturm, P. , Füchtemeier, M. , Foddis, M. , Mueller, S. , Trueman, R. C. , Zille, M. , Rinnenthal, J. L. , Kypraios, T. , Shaw, L. , Dirnagl, U. , & Farr, T. D. (2017). Neuroimaging biomarkers predict brain structural connectivity change in a mouse model of vascular cognitive impairment. Stroke; A Journal of Cerebral Circulation, 48(2), 468–475. 10.1161/strokeaha.116.014394 PMC5266417 28070001

[brb32642-bib-0002] Busza, A. L. , Allen, K. L. , King, M. D. , van Bruggen, N. , Williams, S. R. , & Gadian, D. G. (1992). Diffusion‐weighted imaging studies of cerebral ischemia in gerbils. Potential relevance to energy failure. Stroke; A Journal of Cerebral Circulation, 23(11), 1602–1612. 10.1161/01.str.23.11.1602 1440708

[brb32642-bib-0003] Chavhan, G. B. , Babyn, P. S. , Thomas, B. , Shroff, M. M. , & Haacke, E. M. (2009). Principles, techniques, and applications of T2*‐based MR imaging and its special applications. Radiographics, 29(5), 1433–1449. 10.1148/rg.295095034 19755604 PMC2799958

[brb32642-bib-0004] Chen, Y. , Li, J. , Ma, B. , Li, N. , Wang, S. , Sun, Z. , Xue, C. , Han, Q. , Wei, J. , & Zhao, R. C. (2020). MSC‐derived exosomes promote recovery from traumatic brain injury via microglia/macrophages in rat. Aging (Albany NY), 12(18), 18274–18296. 10.18632/aging.103692 32966240 PMC7585083

[brb32642-bib-0005] Darcourt, J. , Withayasuk, P. , Vukasinovic, I. , Michelozzi, C. , Bellanger, G. , Guenego, A. , Adam, G. , Roques, M. , Januel, A. C. , Tall, P. , Meyrignac, O. , Rousseau, V. , Garcia, C. , Albucher, J. F. , Payrastre, B. , Bonneville, F. , Olivot, J. M. , & Cognard, C. (2019). Predictive value of susceptibility vessel sign for arterial recanalization and clinical improvement in ischemic stroke. Stroke; A Journal of Cerebral Circulation, 50(2), 512–515. 10.1161/strokeaha.118.022912 30602358

[brb32642-bib-0006] de Crespigny, A. J. , Röther, J. , Beaulieu, C. , Moseley, M. E. , & Hoehn, M. (1999). Rapid monitoring of diffusion, DC potential, and blood oxygenation changes during global ischemia. Effects of hypoglycemia, hyperglycemia, and TTX. Stroke; A Journal of Cerebral Circulation, 30(10), 2212–2222. 10.1161/01.str.30.10.2212 10512931

[brb32642-bib-0007] De Jong, G. I. , Farkas, E. , Stienstra, C. M. , Plass, J. R. , Keijser, J. N. , de la Torre, J. C. , & Luiten, P. G. (1999). Cerebral hypoperfusion yields capillary damage in the hippocampal CA1 area that correlates with spatial memory impairment. Neuroscience, 91(1), 203–210. 10.1016/s0306-4522(98)00659-9 10336071

[brb32642-bib-0008] Ferro, D. A. , Mutsaerts, H. J. , Hilal, S. , Kuijf, H. J. , Petersen, E. T. , Petr, J. , Van Veluw, S. J. , Venketasubramanian, N. , Yeow, T. B. , Biessels, G. J. , & Chen, C. (2020). Cortical microinfarcts in memory clinic patients are associated with reduced cerebral perfusion. Journal of Cerebral Blood Flow & Metabolism, 40(9), 1869–1878. 10.1177/0271678x19877403 31558107 PMC7430096

[brb32642-bib-0009] Hazalin, N. , Liao, P. , & Hassan, Z. (2020). TRPM4 inhibition improves spatial memory impairment and hippocampal long‐term potentiation deficit in chronic cerebral hypoperfused rats. Behavioural Brain Research, 393, 112781. 10.1016/j.bbr.2020.112781 32619565

[brb32642-bib-0010] Jin, X. , Li, T. , Zhang, L. , Ma, J. , Yu, L. , Li, C. , & Niu, L. (2017). Environmental enrichment improves spatial learning and memory in vascular dementia rats with activation of Wnt/β‐catenin signal pathway. Medical Science Monitor, 23, 207–215. 10.12659/msm.902728 28082734 PMC5253348

[brb32642-bib-0011] Jing, Z. , Shi, C. , Zhu, L. , Xiang, Y. , Chen, P. , Xiong, Z. , Li, W. , Ruan, Y. , & Huang, L. (2015). Chronic cerebral hypoperfusion induces vascular plasticity and hemodynamics but also neuronal degeneration and cognitive impairment. Journal of Cerebral Blood Flow and Metabolism, 35(8), 1249–1259. 10.1038/jcbfm.2015.55 25853908 PMC4528009

[brb32642-bib-0012] Karasawa, J. , Hashimoto, K. , & Chaki, S. (2008). D‐Serine and a glycine transporter inhibitor improve MK‐801‐induced cognitive deficits in a novel object recognition test in rats. Behavioural Brain Research, 186(1), 78–83. 10.1016/j.bbr.2007.07.033 17854919

[brb32642-bib-0013] Lee, J. M. , Vo, K. D. , An, H. , Celik, A. , Lee, Y. , Hsu, C. Y. , & Lin, W. (2003). Magnetic resonance cerebral metabolic rate of oxygen utilization in hyperacute stroke patients. Annals of Neurology, 53(2), 227–232. 10.1002/ana.10433 12557290

[brb32642-bib-0014] Liu, J. , Jin, D. Z. , Xiao, L. , & Zhu, X. Z. (2006). Paeoniflorin attenuates chronic cerebral hypoperfusion‐induced learning dysfunction and brain damage in rats. Brain Research, 1089(1), 162–170. 10.1016/j.brainres.2006.02.115 16678139

[brb32642-bib-0015] Mamata, H. , Jolesz, F. A. , & Maier, S. E. (2004). Characterization of central nervous system structures by magnetic resonance diffusion anisotropy. Neurochemistry International, 45(4), 553–560. 10.1016/j.neuint.2003.11.014 15186922

[brb32642-bib-0016] Matsuyama, H. , Shindo, A. , Shimada, T. , Yata, K. , Wakita, H. , Takahashi, R. , & Tomimoto, H. (2020). Chronic cerebral hypoperfusion activates AIM2 and NLRP3 inflammasome. Brain Research, 1736, 146779. 10.1016/j.brainres.2020.146779 32171704

[brb32642-bib-0017] Moghaddasi, M. , Taati, M. , Asadian, P. , Khalatbary, A. R. , Asaei, R. , & Pajouhi, N. (2017). The effects of two‐stage carotid occlusion on spatial memory and pro‐inflammatory markers in the hippocampus of rats. The Journal of Physiological Sciences, 67(3), 415–423. 10.1007/s12576-016-0474-z 27470129 PMC10717598

[brb32642-bib-0018] Qi, Y. , Wang, S. , Luo, Y. , Huang, W. , Chen, L. , Zhang, Y. , Liang, X. , Tang, J. , Zhang, Y. , Zhang, L. , Chao, F. , Gao, Y. , Zhu, Y. , & Tang, Y. (2020). Exercise‐induced nitric oxide contributes to spatial memory and hippocampal capillaries in rats. International Journal of Sports Medicine, 41(13), 951–961. 10.1055/a-1195-2737 32643775

[brb32642-bib-0019] Qiao, M. , Malisza, K. L. , Del Bigio, M. R. , & Tuor, U. I. (2002). Transient hypoxia‐ischemia in rats: changes in diffusion‐sensitive MR imaging findings, extracellular space, and Na+‐K+ ‐adenosine triphosphatase and cytochrome oxidase activity. Radiology, 223(1), 65–75. 10.1148/radiol.2231010736 11930049

[brb32642-bib-0020] Sarnyai, Z. , Sibille, E. L. , Pavlides, C. , Fenster, R. J. , McEwen, B. S. , & Toth, M. (2000). Impaired hippocampal‐dependent learning and functional abnormalities in the hippocampus in mice lacking serotonin(1A) receptors. PNAS, 97(26), 14731–14736. 10.1073/pnas.97.26.14731 11121072 PMC18987

[brb32642-bib-0021] Sotak, C. H. (2004). Nuclear magnetic resonance (NMR) measurement of the apparent diffusion coefficient (ADC) of tissue water and its relationship to cell volume changes in pathological states. Neurochemistry International, 45(4), 569–582. 10.1016/j.neuint.2003.11.010 15186924

[brb32642-bib-0022] Vicente, É. , Degerone, D. , Bohn, L. , Scornavaca, F. , Pimentel, A. , Leite, M. C. , Swarowsky, A. , Rodrigues, L. , Nardin, P. , Vieira De Almeida, L. M. , Gottfried, C. , Souza, D. O. , Netto, C. A. , & Gonçalves, C. A. (2009). Astroglial and cognitive effects of chronic cerebral hypoperfusion in the rat. Brain Research, 1251, 204–212. 10.1016/j.brainres.2008.11.032 19056357

[brb32642-bib-0023] Vidyanti, A. N. , Hsieh, J. Y. , Lin, K. J. , Fang, Y. C. , Setyopranoto, I. , & Hu, C. J. (2020). Role of HMGB1 in an animal model of vascular cognitive impairment induced by chronic cerebral hypoperfusion. International Journal of Molecular Sciences, 21(6), 2176. 10.3390/ijms21062176 PMC7139598 32245271

[brb32642-bib-0024] Wang, X. , Cheng, J. L. , Ran, Y. C. , Zhang, Y. , Yang, L. , & Lin, Y. N. (2017). Expression of RGMb in brain tissue of MCAO rats and its relationship with axonal regeneration. Journal of the Neurological Sciences, 383, 79–86. 10.1016/j.jns.2017.10.032 29246630

[brb32642-bib-0025] Wedegärtner, U. , Kooijman, H. , Andreas, T. , Beindorff, N. , Hecher, K. , & Adam, G. (2010). T2 and T2* measurements of fetal brain oxygenation during hypoxia with MRI at 3T: Correlation with fetal arterial blood oxygen saturation. European Radiology, 20(1), 121–127. 10.1007/s00330-009-1513-4 19618188

[brb32642-bib-0026] Williamson, M. R. , Fuertes, C. J. A. , Dunn, A. K. , Drew, M. R. , & Jones, T. A. (2021). Reactive astrocytes facilitate vascular repair and remodeling after stroke. Cell Reports, 35(4), 109048. 10.1016/j.celrep.2021.109048 33910014 PMC8142687

[brb32642-bib-0027] Xi, Y. , Wang, M. , Zhang, W. , Bai, M. , Du, Y. , Zhang, Z. , Li, Z. , & Miao, J. (2014). Neuronal damage, central cholinergic dysfunction and oxidative damage correlate with cognitive deficits in rats with chronic cerebral hypoperfusion. Neurobiology of Learning and Memory, 109, 7–19. 10.1016/j.nlm.2013.11.016 24315928

[brb32642-bib-0028] Yoon, B. , Kim, J. S. , Lee, K. S. , Kim, B. S. , Chung, S. R. , & Kim, Y. I. (2006). Early pathological changes in the cerebellum of patients with pure cerebellar syndrome demonstrated by diffusion‐tensor imaging. European Neurology, 56(3), 166–171. 10.1159/000096181 17035705

[brb32642-bib-0029] Zhang, X. , Shen, X. , Dong, J. , Liu, W.‐C. , Song, M. , Sun, Y. , Shu, H. , Towse, C.‐L. , Liu, W. , Liu, C.‐F. , & Jin, X. (2019). Inhibition of reactive astrocytes with fluorocitrate ameliorates learning and memory impairment through upregulating CRTC1 and synaptophysin in ischemic stroke rats. Cellular and Molecular Neurobiology, 39(8), 1151–1163. 10.1007/s10571-019-00709-0 31270712 PMC11452224

[brb32642-bib-0030] Zuloaga, K. L. , Zhang, W. , Yeiser, L. A. , Stewart, B. , Kukino, A. , Nie, X. , Roese, N. E. , Grafe, M. R. , Pike, M. M. , Raber, J. , & Alkayed, N. J. (2015). Neurobehavioral and imaging correlates of hippocampal atrophy in a mouse model of vascular cognitive impairment. Translational Stroke Research, 6(5), 390–398. 10.1007/s12975-015-0412-z 26040424 PMC4561019

